# Combined effect of established BMI loci on obesity-related traits in an Algerian population sample

**DOI:** 10.1186/s12863-014-0128-1

**Published:** 2014-11-25

**Authors:** Manel Nassima Badsi, Sounnia Mediene-Benchekor, Hadjira Ouhaibi-Djellouli, Sarah Aicha Lardjam-Hetraf, Houssam Boulenouar, Djabaria Naïma Meroufel, Xavier Hermant, Imane Hamani-Medjaoui, Nadhira Saidi-Mehtar, Philippe Amouyel, Leila Houti, Aline Meirhaeghe, Louisa Goumidi

**Affiliations:** Laboratoire de Génétique Moléculaire et Cellulaire, Université des Sciences et de Technologie d’Oran Mohamed Boudiaf, BP 1505 El M’Naouar, 31036 Oran, Algeria; Département de Biotechnologie, Faculté des Sciences de la Nature et de la Vie, Université d’Oran, BP 1524. El M’Naouar, 31000 Oran, Algeria; INSERM, U744; Institut Pasteur de Lille, Université Lille Nord de France, 1 rue du Pr. Calmette, BP 245, F-59019, Lille Cedex, France; CNAS Hai Bouamama (El Hassi), Caisse Nationale des Assurances Sociales des travailleurs salariés, Clinique Spécialisée en Orthopédie et Rééducation des Victimes des Accidents de Travail, Oran, Algeria; Faculté de Médecine, Université Djillali Liabes de Sidi Bel Abbès, BP 89, 22000, Sidi-Bel-Abbès, Algeria; LABoratoire des Systèmes d’Information en Santé, Université d’Oran, BP 1524 El M’Naouar, 31000 Oran, Algeria

**Keywords:** Genetic predisposition score, Polymorphism, BMI, Obesity, Algerian population, ISOR study

## Abstract

**Background:**

Genome-wide association studies have identified variants associated with BMI in populations of European descent. We sought to establish whether genetic variants that are robustly associated with BMI could modulate anthropometric traits and the obesity risk in an Algerian population sample, the ISOR study.

The ISOR study of 787 adult subjects (aged between 30 and 64) provided a representative sample of the population living in the city of Oran (north-west of Algeria). We investigated the combined effect of 29 BMI established genetic variants using a genetic predisposition score (GPS) on anthropometric traits and obesity risk in 740 subjects.

**Results:**

We found that each additional risk allele in the GPS was associated with an increment in the mean [95% CI] for BMI of 0.15 [0.06 - 0.24] kg/m^2^ (*p* = 0.001). Although the GPS was also associated with higher waist (*p* = 0.02) and hip (*p* = 0.02) circumferences, these associations were in fact driven by BMI. The GPS was also associated with an 11% higher risk of obesity (OR [95%CI] = 1.11 [1.05 - 1.18], *p* = 0.0004).

**Conclusions:**

Our data showed that a GPS comprising 29 BMI established loci developed from Europeans seems to be a valid score in a North African population. Our findings contribute to a better understanding of the genetic susceptibility to obesity in Algeria.

**Electronic supplementary material:**

The online version of this article (doi:10.1186/s12863-014-0128-1) contains supplementary material, which is available to authorized users.

## Background

Obesity (as characterized by excess body fat) is an established risk factor for cardiovascular and metabolic diseases. Indeed, each unit increase in the body mass index (BMI) increases the risk of hypertension by a factor of 5 and the risks of coronary artery disease and stroke by a factor of 3.6 [1]. It is noteworthy that 80% of people with type 2 diabetes are obese [2]. Moreover, non-alcoholic steatohepatitis, impotency, infertility, and several types of cancer have all been linked to obesity [1]. Overall, obesity increases the risk of premature death. Obesity has reached epidemic proportions in modern society [3]. In 2008, more than 500 million adults worldwide were considered to be clinically obese [4]. However, the prevalence of obesity and overweight differs from one region of the world to another, with 33.5% in the USA [5], 23.1% in Canada [6], between 5.1% and 32.4% in Europe (with a west/east/south gradient) [7], 14.9% in Morocco [8], 29.6% in Tunisia [9] and 21.2% in Algeria [10]. Obesity is increasingly prevalent in many African countries and other developing countries undergoing nutritional transitions as a result of urbanization, demographic transitions and the adoption of Western lifestyles [11].

Common obesity is caused by the interaction of multiple genetic and environmental factors; with a heritability ranging from 40 to 70% [12]. Genetic approaches have improved our understanding of the biological bases of obesity. To date, genome-wide association studies (GWASs) have identified and confirmed 32 BMI loci in European populations [13-19]. The replication of association signals in independent populations is a mandatory approach for characterizing gene-disease relationships [20]. Because initial and replication studies have been mainly reported in populations of European descent, the challenge remains to extend the studies to other populations [20]. Moreover, as each individual single nucleotide polymorphism (SNP) exerts a moderate genetic effect and thus explains a small proportion of the total BMI variation, the analysis of the combined effect of sets of variants (by calculating a genetic predisposition score (GPS)) [21] appears necessary and also useful in moderate sample size population samples which are underpowered compared to GWASs.

In the present study, we assessed whether a GPS for BMI developed from Europeans is a valid score in a North African population. Although a few association studies have been performed in this part of the world [22-26], no one examined the combined set of established BMI loci. Therefore, we tested whether the combination of variants robustly associated with BMI in many European populations also influence anthropometric traits and obesity risk in an Algerian population sample (provided by the ISOR study).

## Methods

### The ISOR (*InSulino*-*résistance à ORan*) study

The cross-sectional population-based ISOR study was performed from 2007 to 2009. The study’s objectives and procedures were approved by the independent ethics committee at the Algerian National Agency for the Development of Health Research (since renamed as the Thematic Agency of Research in Health Sciences). The ISOR study was a population-based, cross-sectional study of a representative sample of 787 subjects (378 men and 409 women, aged between 30 and 64) recruited from within the city of Oran (north-west of Algeria). Subjects were selected at random from social security rolls. All subjects consented freely to participation in the study. Details of the studies have been described elsewhere [27].

A questionnaire on lifestyle (physical activity, tobacco use and alcohol intake), personal and family medical histories, current medication, socio-economic status and educational level was completed during a face-to-face interview. The ISOR questionnaire lifestyle was an adapted version of the one used in the STEP study (investigation Stepwise led in Algeria in 2003) which was validated by the WHO. The level of physical activity was defined in quartiles as “none”, “low”, “medium” and “high” after summing exercise scores for sporting activities, walking, housework and physical activity at work.

The anthropometric measurements included height, body weight, waist and hip circumferences. Height and weight were measured while the subject was barefoot and lightly dressed. The BMI was calculated according to the Quetelet equation. Normal weight was considered for BMI < 25 kg/m^2^, overweight was considered for 25 ≤ BMI < 30 kg/m^2^ and obesity was considered for BMI ≥ 30 kg/m^2^.

Genomic DNA was extracted from white blood cells by using the Stratagene® kit (Agilent Technologies, Les Ulis, France), according to the manufacturer’s protocol.

### SNP selection and genotyping

We selected 32 SNPs known to be associated with BMI [19] within or near the following genes: *FTO*, *MC4R*, *TMEM18*, *GNPDA2*, *BDNF*, *NEGR1*, *SH2B1*, *ETV5*, *MTCH2*, *KCTD15*, *TFAP2B*, *NRXN3*, *FAIM2*, *SEC16B*, *RBJ*-*ADCY3*-*POMC*, *GPRC5B*, *MAP2K5*-*LBXCOR1*, *QPCTL*-*GIPR*, *TNNI3K*, *SLC39A8*, *FLJ35779*-*HMGCR*, *LRRN6C*, *TMEM160*, *FANCL*, *CADM2*, *PRKD1*, *LRP1B*, *PTBP2*, *MTIF3*-*GTF3A*, *RPL27A*-*TUB*, *NUDT3*-*HMGA1* and *ZNF608*.

Genotyping was performed using KASPar technology (KBioscience, Hoddesdon, UK). The genotyping of the *ZNF608* rs4836133 SNP failed. The genotyping success rates of the *FANCL* rs887912 and *CADM2* rs13078807 SNPs were too low (75% and 76%, respectively) to be considered in the analyses. The genotyping success rate of the 29 other SNPs was at least 95%. So 29 SNPs were considered in the final analyses.

### Statistical analysis

Statistical analyses were performed with SAS software (version 9.1, SAS Institute Inc., Cary, NC, USA). Deviation from the Hardy-Weinberg equilibrium was tested using a χ^2^ test and the threshold for statistical significance was set to *p* ≤ 0.0017 (with Bonferroni correction for 29 independent SNPs at α = 0.05).

The GPS was obtained as previously described [28]. Briefly, a weighting method was used to calculate the GPS on the basis of 29 genotyped SNPs. The genotypes were coded as 0, 1, or 2 according to the number of copies of the effect allele. Each SNP was weighted according to its relative effect size (i.e. the β coefficient); in order to measure the effect of each SNP on BMI with greater accuracy and precision, β coefficients were derived from Speliotes *et al*. [19]. The GPS was calculated by multiplying each β-coefficient by the number of corresponding risk alleles and then summing the products. Because this produced a score out of 8.24 (twice the sum of the reported β-coefficients), all values were divided by 8.24 and multiplied by 58 (number of alleles) to make the GPS easier to interpret: each point of the GPS corresponds to one risk allele. When calculating the GPS, missing genotype data were replaced with the average allele count for the corresponding SNP. However, 47 individuals with missing genotypes for more than 3 SNPs were excluded from the GPS analyses.

Intergroup comparisons of means were performed with a general linear model using an additive genetic model. A Pearson χ^2^ test was used to compare groups in terms of genotype and allele distributions.

Odds ratios (ORs) were obtained by multivariate logistic regression analyses with an additive genetic model.

For BMI and obesity risk, the covariables were age, gender, smoking status and physical activity. For waist circumference and hip circumference, the covariables were age, gender, smoking status, physical activity and (in some models) BMI.

For individuals associations, after Bonferroni correction, only individuals associations with a *p* value below 0.0017 were considered to be statistically significant (i.e. 0.05 divided by 29 polymorphisms).

For combined association (GPS analyses); statistical significance was set to *p* ≤ 0.05. Power calculations for individual SNP analyses were performed using Quanto v1.2.4 (http://biostats.usc.edu/Quanto.html) using a one-sided p value at 0.05.

Concerning the GPS analysis, the statistical power of detecting a significant association with BMI was 98% (calculated with the pwr R package developed by Stéphane Champely).

## Results

### Characteristics of the study subjects

The ISOR study includes 340 (43.2%) normal weight subjects, 280 (35.6%) overweight subjects and 167 (21.2%) obese subjects (Additional file 1).

### Distributions and individual associations with anthropometric traits

All 29 SNPs conformed to Hardy-Weinberg equilibrium (Table 1). When we compared allelic frequencies of these 29 loci between the ISOR study and the study of Speliotes *et al*., the allelic distributions differed for 5 SNPs (Table 1). For example, for *SH2B1* rs7359397, the T allele frequency was 0.15 and 0.40 in the ISOR and Speliotes *et al*.’ studies, respectively.

**Table 1:** **Distributions of the 29 genotyped SNPs in the ISOR study**

		**ISOR study**							**Speliotes** ***et al.*** **study**
**Nearby gene or locus**	**SNP**	**EA**	**OA**	**Genotype**			***p HWE***	**EAF/OAF**	**EAF/OAF**
				**0**	**1**	**2**			
*FTO*	rs9939609	A	T	278(0.37)	370(0.49)	106(0.14)	0.33	0.39/0.61	0.42/0.58
*TMEM18*	rs2867125	C	T	21(0.03)	212(0.28)	520(0.69)	0.96	0.83/0.17	0.83/0.17
*MC4R*	rs571312	A	C	454(0.60)	267(0.36)	31(0.04)	0.29	0.22/0.78	0.24/0.76
*GNPDA2*	rs10938397	G	A	256(0.35)	380(0.51)	107(0.14)	0.08	0.40/0.60	0.43/0.57
*BDNF*	rs10767664	A	T	31(0.04)	219(0.29)	496(0.67)	0.29	0.81/0.19	0.78/0.22
*NEGR1*	rs2815752	A	G	69(0.09)	299(0.41)	372(0.50)	0.43	0.70/0.30	0.61/0.39
*SH2B1*	rs7359397	T	C	547(0.73)	186(0.25)	17(0.02)	0.82	0.15/0.85	0.40/0.60
*ETV5*	rs9816226	T	A	40(0.05)	252(0.34)	448(0.61)	0.59	0.78/0.22	0.82/0.18
*MTCH2*	rs3817334	T	C	260(0.35)	335(0.44)	160(0.21)	7.5×10^−3^	0.43/0.57	0.41/0.59
*KCTD15*	rs29941	G	A	59(0.08)	284(0.37)	417(0.55)	0.25	0.74/0.26	0.67/0.33
*SEC16B*	rs543874	G	A	522(0.69)	220(0.29)	18(0.02)	0.41	0.17/0.83	0.19/0.81
*TFAP2B*	rs987237	G	A	552(0.73)	176(0.23)	28(0.04)	4.0×10^−3^	0.15/0.85	0.18/0.82
*FAIM2*	rs7138803	A	G	359(0.48)	319(0.42)	73(0.10)	0.87	0.31/0.69	0.38/0.62
*NRXN3*	rs10150332	C	T	455(0.61)	256(0.34)	38(0.05)	0.76	0.22/0.78	0.21/0.79
*RBJ*	rs713586	C	T	203(0.27)	375(0.51)	163(0.22)	0.74	0.47/0.53	0.47/0.53
*GPRC5B*	rs12444979	C	T	8(0.01)	144(0.19)	607(0.80)	0.89	0.89/0.11	0.87/0.13
*MAP2K5*	rs2241423	G	A	58(0.08)	274(0.36)	419(0.56)	0.19	0.74/0.26	0.78/0.22
*QPCTL*	rs2287019	C	T	34(0.05)	223(0.30)	484(0.65)	0.19	0.80/0.20	0.80/0.20
*TNNI3K*	rs1514175	A	G	229(0.30)	358(0.47)	170(0.23)	0.19	0.46/0.54	0.43/0.57
*SLC39A8*	rs13107325	T	C	730(0.96)	33(0.04)	-	0.54	0.02/0.98	0.07/0.93
*FLJ35779*	rs2112347	T	G	78(0.11)	298(0.40)	364(0.49)	0.15	0.69/0.31	0.63/0.37
*LRRN6C*	rs10968576	G	A	590(0.78)	147(0.19)	19(0.03)	7.3×10^−3^	0.12/0.88	0.31/0.69
*TMEM160*	rs3810291	A	G	209(0.28)	356(0.47)	190(0.25)	0.16	0.49/0.51	0.67/0.33
*PRKD1*	rs11847697	T	C	569(0.76)	166(0.22)	17(0.02)	0.21	0.13/0.87	0.04/0.96
*LRP1B*	rs2890652	C	T	526(0.69)	212(0.28)	19(0.03)	0.68	0.17/0.83	0.18/0.82
*PTBP2*	rs1555543	C	A	238(0.32)	378(0.51)	129(0.17)	0.32	0.43/0.57	0.59/0.41
*MTIF3*	rs4771122	G	A	500(0.66)	225(0.30)	27(0.04)	0.74	0.19/0.81	0.24/0.76
*RPL27A*	rs4929949	C	T	277(0.37)	325(0.44)	142(0.19)	8.4×10^−3^	0.41/0.59	0.52/0.48
*NUDT3*	rs206936	G	A	344(0.46)	323(0.43)	79(0.11)	0.73	0.32/0.68	0.21/0.79

*EA*: effect allele. *OA*: other allele. *EAF*: effect allele frequency. *OAF*: other allele frequency.

Genotypes were coded as 0/1/2, indicating the subject’s number of copies of the designated effect alleles.

*p HWE*: p values for Hardy-Weinberg Equilibrium test.

Power calculations indicated that, based on results from Speliotes *et al*. [19], the statistical power of our study to detect significant associations between individual SNPs and BMI was below 44%. Nevertheless, the *RBJ* rs713586 and *RPL27A* rs4929949 SNPs were nominally associated with BMI (β = 0.62 ± 0.25, *p* = 0.01, and β = 0.67 ± 0.24, *p* = 0.006, respectively); and 23 of the 29 tested SNPs were directionally consistent with the results reported in the original GWAS on BMI (Additional file 2). This result was greater than that expected by chance (Binomial test, *p* = 0.0009).

### Combined associations with anthropometric-related traits and obesity risk

The 29 SNPs were used to calculate the GPS, which was normally distributed (mean: 25.7 ± 3.7 alleles; range: 14.2 to 37.6 alleles). We observed significant associations between the GPS and BMI, waist circumference and hip circumference (Table 2). The mean [95% CI] allele effect of the GPS was +0.15 [0.06 - 0.24] kg/m^2^ (*p* = 0.001) for BMI, +0.26 [0.03 - 0.49] cm (*p* = 0.02) for waist circumference and +0.22 [0.04 - 0.40] cm (*p* = 0.02) for hip circumference. We did not detect a statistically significant association between the GPS and the waist-to-hip ratio (*p* = 0.37). Associations with waist circumference and hip circumference were no longer statistically significant after further adjustment for BMI. Of note, when the analyses did not take into account missing genotypes, similar results were obtained for BMI, waist circumference, hip circumference and the waist-to-hip ratio (data not shown).

**Table 2:** **Effect of the genetic predisposition score on anthropometric variables in the ISOR study** (**n** = **740**)

			**SE**	**LCL**	**UCL**	***p***	p*
BMI (kg/m^2^)		0.15	0.05	0.06	0.24	0.001	-
Waist (cm)		0.26	0.12	0.03	0.49	0.02	0.23
Hip (cm)		0.22	0.09	0.04	0.40	0.02	0.60
Waist-to-hip ratio		0.001	0.001	−0.001	0.002	0.37	0.55

The β coefficients represent the effect sizes.

*SE*: standard error; *LCL*: lower confidence limit; *UCL*: upper confidence limit.

*p* values were adjusted for age, gender, physical activity and smoking status.

*p values were adjusted for age, gender, physical activity, smoking status and BMI.

To distinguish between the effects of the GPS and the effects of the covariables classically associated with BMI (age, gender, physical activity and smoking status), we compared the crude and adjusted models (Table 3). The GPS alone accounted for 1.0% of the BMI variance and the covariables accounted for 13.1% of the variance. Overall, the GPS and covariables explained 14.1% of the BMI variance.

**Table 3:** **Effects of the genetic predisposition score and covariables on BMI in the ISOR study** (**n** = **740**)

**Models**	**β**	**SE**	**LCL**	**UCL**	***p***	**Explained variance (%)**
Model 1	0.14	0.05	0.04	0.24	0.006	1.0
Model 2	0.15	0.05	0.06	0.24	0.001	14.1

The β coefficients represent the effect sizes.

*SE*: standard error; *LCL*: lower confidence limit; *UCL*: upper confidence limit.

Model 1: crude *p* value.

Model 2: *p* value adjusted for age, gender, physical activity and smoking status.

Next, we investigated the association between the GPS and the obesity risk. Because, overweight participants could not be considered as obese or normal weight subjects, we removed them for the analyses. We detected a significant association between the GPS and the obesity risk (OR [95%CI] = 1.11 [1.05 - 1.18], *p* = 0.0004). We also examined the association between the obesity risk and the GPS in quartiles (Table 4). Subjects in the highest GPS quartile (i.e. > 28.3 alleles) had a higher obesity risk than subjects in the lowest GPS quartile (i.e. < 23.2 alleles) (OR = 2.53 [1.38 - 4.65], *p* = 0.003).

**Table 4:** **The obesity risk per quartile of the genetic predisposition score in the ISOR study**, **excluding overweight subjects**

**GPS** **(number of alleles)**	**Normal weight subjects** **/obese subjects** **(n)**	***p*** **trend**	**OR**	**LCL**	**UCL**	***p***
<23.2	91/33	0.0003	reference			
[23.2 - 25.6]	87/33		0.88	0.46	1.67	0.69
[25.6 - 28.3]	71/36		1.62	0.82	3.13	0.16
>28.3	71/52		2.53	1.38	4.65	0.003

*p* values were adjusted for age, gender, physical activity and smoking status.

*LCL*: lower confidence limit; *UCL*: upper confidence limit.

As the rs3817334, rs987237, rs10968576, rs4929949 SNPs could be considered as outliners in terms of respect of the Hardy-Weinberg equilibrium (4.0×10^−3^ ≤ p ≤ 8.4×10^−3^), we performed all GPS analyses without these 4 SNPs and similar results were obtained (data not shown).

## Discussion

Meta-analyses of GWASs have identified 32 loci as being unequivocally associated with BMI, individually or combined in a genetic predisposition score in populations of European descent [19]. In the present study, we have investigated the combined effects of 29 successfully genotyped, established BMI loci on anthropometric variables and obesity risk in a sample of the general population from north-west of Algeria (n = 740).

The prevalence of obesity in the ISOR study (21.2%) is similar to that reported in Algeria (21.2%) in the Transition and Health Impact In North Africa (TAHINA) study in 2010 [10]. The prevalence of obesity in Oran is higher than in France (14.5%) [29], Spain (13.6%) [30] and Italy (8.2%) [31], lower than in the USA (33.5%) [5], Canada (23.1%) [6] and the UK (23.5%) [32], and similar to that in Nigeria (21.2%) [33]. When compared with other urban North African populations, the prevalence of obesity in Oran is higher than in Morocco (14.9%) [8] and lower than in Tunisia (29.6%) [9].

Although the ISOR study was not sufficiently powered (<44%) to detect significant individual associations, most of the tested SNPs presented effects with the expected direction (23 of the 29 tested). We also observed that few SNPs presented significant allele frequency differences between the ISOR and Speliotes *et al*. [19] studies. Differences in allele frequencies may contribute to differences in disease prevalence between ethnic groups [34].

Although a few association studies on BMI or obesity have been performed in North African samples [22-26], no one examined the GWAS established BMI loci either individually or combined. In the ISOR study, the GPS (corresponding to the 29 established, BMI-associated SNPs’ cumulative contribution) for which we had 98% power in the ISOR study, showed a significant, positive association with BMI (*p* = 0.001). Each additional BMI-raising allele was associated with a mean increment of 0.15 kg/m^2^ (which corresponds to a weight increment of 434 g for a person measuring 1.70 m in height). Although the GPS was also significantly associated with waist and hip circumferences, these associations were mediated by BMI.

The overall known genetic susceptibility associated with the GPS explained only 1.0% of the variance in BMI, whereas the combined effect of genetic and known environmental factors accounted for 14.1%. Our results are in agreement with previous reports [19,35]. Moreover, the GPS was associated with a 11% higher obesity risk and subjects with more than 28.3 risk alleles had a 2.5-fold higher risk of obesity. Although (i) it has been calculated that genetic factors account for between 40% and 70% of the population variation in BMI and (ii) the 29 SNPs studied here have been robustly validated as BMI-susceptible variants in GWA/replication studies, their combined effect on BMI and obesity risk was quite small. However, our results are in agreement with previous reports [16,19,35-39].

## Conclusion

In conclusion, although larger samples will be needed to firmly replicate our findings, our data showed that a GPS comprising 29 BMI established loci in Europeans was associated with higher BMI and obesity risk in an Algerian population. Our findings contribute to a better understanding of the genetic susceptibility to obesity in Algeria.

## Additional files

Additional file 1
**Clinical characteristics of the subjects in the ISOR study.**
Additional file 2
**Individual associations between the 29 genotyped SNPs and anthropometric parameters in the ISOR study.**

## Abbreviations

BMI: Body mass index
CI: Confidence interval
GPS: Genetic predisposition score
GWAS: Genome-wide association Study
SE: Standard error
SNP: Single-nucleotide polymorphism
OR: Odds ratio

## References

[1] Kopelman P (2007). Health risks associated with overweight and obesity. Obes Rev.

[2] Bloomgarden ZT (2000). American Diabetes Association Annual Meeting, 1999: diabetes and obesity. Diabetes Care.

[3] Yancey AK, McCarthy WJ, Taylor WC, Merlo A, Gewa C, Weber MD, Fielding JE (2004). The Los Angeles Lift Off: a sociocultural environmental change intervention to integrate physical activity into the workplace. Prev Med.

[4] World Health Organisation (2012). World health statistics.

[5] Flegal KM, Carroll MD, Ogden CL, Johnson CL (2002). Prevalence and trends in obesity among US adults, 1999–2000. JAMA.

[6] Shields M: **Nutrition: Findings from the Canadian Community Health Survey – Overweight Canadian children and adolescents.** *Component of Statistics Canada Catalogue* 2005, no.82–620-MWE2005001.

[7] Berghöfer A, Pischon T, Reinhold T, Apovian CM, Sharma AM, Willich SN (2008). Obesity prevalence from a European perspective: a systematic review. BMC Public Health.

[8] El Rhazi K, Nejjari C, Zidouh A, Bakkali R, Berraho M, Barberger-Gateau P (2010). Prevalence of obesity and associated sociodemographic and lifestyle factors in Morocco. Public Health Nutr.

[9] El Ati J, Traissac P, Delpeuch F, Aounallah-Skhiri H, Beji C, Eymard-Duvernay S, Bougatef S, Kolsteren P, Maire B, Ben Romdhane H (2012). Gender Obesity Inequities Are Huge but Differ Greatly According to Environment and Socio-Economics in a North African Setting: A National Cross-Sectional Study in Tunisia. PLoS One.

[10] Atek M, Laid Y, Mezimeche N, Boutekdjiret L, Lebcir H: **L’Obésité chez l’adulte de 35 à 70 ans en Algérie. Projet TAHINA.** *Institut national de santé publique Alger Algérie* 2010, 1–93.

[11] Levitt NS (2008). Diabetes in Africa: epidemiology, management and healthcare challenges. Heart.

[12] Atwood LD, Heard-costa NL, Cupples LA, Jaquish CE, Wilson PWF, D’Agostino RB (2002). Genome wide linkage analysis of body mass index across 28 years of the Framingham Heart Study. Am J Hum Genet.

[13] Frayling TM, Timpson NJ, Weedon MN, Zeggini E, Freathy RM, Lindgren CM, Perry JRB, Elliott KS, Lango H, Rayner NW, Shields B, Harries LW, Barrett JC, Sian E, Groves CJ, Knight B, Patch AM, Ness AR, Ebrahim S, Lawlor DA, Ring SM, Ben-Shlomo Y, Jarvelin MR, Sovio U, Bennett AJ, Melzer D, Ferrucci L, Loos RJF, Barroso I, Wareham NJ (2007). A Common Variant in the FTO Gene Is Associated with Body Mass Index and Predisposes to Childhood and Adult Obesity. Science.

[14] Loos RJF, Lindgren CM, Li S, Wheeler E, Hua J, Prokopenko I, Inouye M, Freathy RM, Attwood AP, Beckmann JS, Berndt SI, Bergmann S, Bennett AJ, Bingham SA, Bochud M, Brown M, Cauchi S, Connell JM, Cooper C, Smith GD, Day I, Dina C, De S, Dermitzakis ET, Doney ASF, Elliott KS, Elliott P, Evans DM, Farooqi IS, The Prostate, Lung, Colorectal, and Ovarian (PLCO) Cancer Screening Trial (2008). Common variants near MC4R are associated with fat mass, weight and risk of obesity. Nat Genet.

[15] Thorleifsson G, Walters GB, Gudbjartsson DF, Steinthorsdottir V, Sulem P, Helgadottir A, Styrkarsdottir U, Gretarsdottir S, Thorlacius S, Jonsdottir I, Jonsdottir T, Olafsdottir EJ, Olafsdottir GH, Jonsson T, Jonsson F, Borch-Johnsen K, Hansen T, Andersen G, Jorgensen T, Lauritzen T, Aben KK, Verbeek ALM, Roeleveld N, Kampman E, Yanek LR, Becker LC, Tryggvadottir L, Rafnar T, Becker DM, Gulcher J (2009). Genome-wide association yield new sequence variants at seven loci that associate with measures of obesity. Nat Genet.

[16] Willer CJ, Speliotes EK, Loos RJF, Lindgren CM, Heid IM, Berndt SI, Elliott AL, Jackson AU, Lamina C, Lettre G, Lim N, Lyon HN, McCarroll SA, Papadakis K, Qi L, Randall JC, Roccasecca RM, Sanna S, Scheet P, Weedon MN, Wheeler E, Zhao JH, Jacobs LC, Prokopenko I, Soranzo N, Tanaka T, Timpson NJ, Almgren P, Bennett A, Bergman RN (2009). Six new loci associated with body mass index highlight a neuronal influence on body weight regulation. Nat Genet.

[17] Heard-Costa LN, Zillikens MC, Monda KL, Johansson A, Harris TB, Fu M, Haritunians T, Feitosa MF, Aspelund T, Eiriksdottir G, Garcia M, Launer LJ, Smith AV, Mitchell BD, McArdle PF, Shuldiner AR, Bielinski SJ, Boerwinkle E, Brancati F, Demerath EW, Pankow JS, Arnold AM, Chen YI, Glazer NL, McKnight B, Psaty BM, Rotter JI, Amin N, Campbell H, Gyllensten U (2009). NRXN3 is a novel locus for waist circumference: a genome-wide association study from the CHARGE Consortium. PLoS Genet.

[18] Lindgren CM, Heid IM, Randall JC, Lamina C, Steinthorsdottir V, Qi L, Speliotes EK, Thorleifsson G, Willer CJ, Herrera BM, Jackson AU, Lim N, Scheet P, Soranzo N, Amin N, Aulchenko YS, Chambers JC, Drong A, Luan J, Lyon HN, Rivadeneira F, Sanna S, Timpson NJ, Zillikens MC, Zhao JH, Almgren P, Bandinelli S, Bennett AJ, Bergman RN, Bonnycastle LL (2009). Genome-wide association scan meta-analysis identifies three loci influencing adiposity and fat distribution. PLoS Genet.

[19] Speliotes EK, Willer CJ, Berndt SI, Monda KL, Thorleifsson G, Jackson AU, Lango AH, Lindgren CM, Luan J, Magi R, Randall JC, Vedantam S, Winkler TW, Qi L, Workalemahu T, Heid IM, Steinthorsdottir V, Stringham HM, Weedon MN, Wheeler E, Wood AR, Ferreira T, Weyant RJ, Segre AV, Estrada K, Liang L, Nemesh J, Park JH, Gustafsson S, Kilpelainen TO (2010). Association analyses of 249,796 individuals reveal 18 new loci associated with body mass index. Nat Genet.

[20] Chanock SJ, Manolio T, Boehnke M, Boerwinkle E, Hunter DJ, Thomas G, Hirschhorn JN, Abecasis G, Altshuler D, Bailey-Wilson JE, Brooks LD, Cardon LR, Daly M, Donnelly P, Fraumeni JF, Freimer NB, Gerhard DS, Gunter C, Guttmacher AE, Guyer MS, Harris EL, Hoh J, Hoover R, Kong CA, Merikangas KR, Morton CC, Palmer LJ, Phimister EG, Rice JP, NCI-NHGRI Working Group on Replication in Association Studies (2007). Replicating genotype-phenotype associations. Nature.

[21] Loos RJ (2012). Genetic determinants of common obesity and their value in prediction. Best Pract Res Clin Endocrinol Metab.

[22] Bouhaha R, Baroudi T, Ennafaa H, Emanuel V, Hafawa A, Sassi R, Vatin V, Froguel P, Benammar-el Gaaied A, Meyre D, Vaxillaire M (2010). Study of TNFalpha -308G/A and IL6–174G/C polymorphisms in type 2 diabetes and obesity risk in the Tunisian population. Clin Biochem.

[23] Bouhaha R, Choquet H, Meyre D, Abid Kamoun H, Ennafaa H, Baroudi T, Sassi R, Vaxillaire M, Elgaaied A, Froguel P, Cauchi S (2010). TCF7L2 is associated with type 2 diabetes in nonobese individuals from Tunisia. Pathol Biol (Paris).

[24] Boumaiza I, Omezzine A, Rejeb J, Rebhi L, Ouedrani A, Ben Rejeb N, Rebhi L, Ouedrani A, Ben Rejeb N, Nabli N, Ben Abdelaziz A, Bouslama A (2012). Relationship between leptin G2548A and leptin receptor Q223R gene polymorphisms and obesity and metabolic syndrome risk in Tunisian volunteers. Genet Test Mol Biomarkers.

[25] Boumaiza I, Omezzine A, Rejeb J, Rebhi L, Ben Rejeb N, Nabli N, Ben Abdelaziz A, Bouslama A (2012). Association between four resistin polymorphisms, obesity, and metabolic syndrome parameters in tunisian volunteers. Genet Test Mol Biomarkers.

[26] El Achhab Y, Meyre D, Bouatia-Naji N, Berraho M, Deweirder M, Vatin V, Delplanque J, Serhier Z, Lyoussi B, Nejjari C, Froguel P, Chikri M (2009). Association of the ENPP1 K121Q polymorphism with type 2 diabetes and obesity in the Moroccan population. Diabetes Metab.

[27] Boulenouar H, Mediene Benchekor S, Meroufel DN, Lardjam Hetraf SA, Ouhaibi Djellouli H, Hermant X, Grenier-Boley B, Hamani Medjaoui I, Saidi Mehtar N, Amouyel P, Houti L, Meirhaeghe A, Goumidi L (2013). Impact of APOE gene polymorphisms on the lipid profile in an Algerian population. Lipids Health Dis.

[28] Qi Q, Chu AY, Kang JH, Jensen MK, Curhan GC, Pasquale LR, Ridker PM, Hunter DJ, Willett WC, Rimm EB, Chasman DI, Hu FB, Qi L (2012). Sugar-sweetened beverages and genetic risk of obesity. N Engl J Med.

[29] Charles MA, Eschwege E, Basdevant A (2008). Monitoring the obesity epidemic in France. The Obepi surveys 1997–2006. Obesity.

[30] Rodríguez-Rodríguez E, López-Plaza B, López-Sobaler AM, Ortega RM (2011). Overweight and obesity among Spanish adults. Nutr Hosp.

[31] Gallus S, Colombo P, Scarpino V, Zuccaro P, Negri E, Apolone G, La Vecchia C (2006). Overweight and obesity in Italian adults 2004, and an overview of trends since 1983. Eur J Clin Nutr.

[32] British Heart Foundation (2006). Diet, Physical Activity and Obesity Statistics.

[33] Wahab KW, Sani MU, Yusuf BO, Gbadamosi M, Gbadamosi A, Yandutse MI (2011). Prevalence and determinants of obesity - a cross-sectional study of an adult Northern Nigerian population. Int Arch Med.

[34] Cross DS, Ivacic LC, Stefanski EL, McCarty CA (2010). Population based allele frequencies of disease associated polymorphisms in the Personalized Medicine Research Project. BMC Genet.

[35] Goumidi L, Cottel D, Dallongeville J, Amouyel P, Meirhaeghe A (2014). Effects of established BMI-associated loci on obesity-related traits in a French representative population sample. BMC Genet.

[36] Hoed MD, Ekelund U, Brage S, Grontved A, Zhao JH, Sharp SJ, Ong KK, Wareham NJ, Loos RJF (2010). Genetic Susceptibility to Obesity and Related Traits in Childhood and Adolescence. Diabetes.

[37] Cuypers KF, Loos RJF, Kvaløy K, Kulle B, Romundstad P, Holmen TL (2012). Obesity-Susceptibility Loci and Their Influence on Adiposity-Related Traits in Transition from Adolescence to Adulthood - The HUNT Study. PLoS One.

[38] van Vliet-Ostaptchouk JV, den Hoed M, Luan J, Zhao JH, Ong KK, Van Der Most PJ, Wong A, Hardy R, Kuh D, van der Klauw MM, Bruinenberg M, Khaw KT, Wolffenbuttel BHR, Wareham NJ, Snieder H, Loos RJF (2013). Pleiotropic effects of obesity-susceptibility loci on metabolic traits: a meta-analysis of up to 37,874 individuals. Diabetologia.

[39] Berndt SI, Gustafsson S, Mägi R, Ganna A, Wheeler E, Feitosa MF, Justice AE, Monda KL, Croteau-Chonka DC, Day FR, Esko T, Fall T, Ferreira T, Gentilini D, Jackson AU, Luan J, Randall JC, Vedantam S, Willer CJ, Winkler TW, Wood AR, Workalemahu T, Hu YJ, Lee SH, Liang L, Lin DY, Min JL, Neale BM, Thorleifsson G, Yang J (2013). Genome-wide meta-analysis identifies 11 new loci for anthropometric traits and provides insights into genetic architecture. Nat Genet.

