# Supplementary material for: Combined effect of established BMI loci on obesity-related traits in an Algerian population sample
Source: BMC Genet. 2014 Nov 25;15:128. doi: 10.1186/s12863-014-0128-1 (PMC4247883; doi:10.1186/s12863-014-0128-1)
Supplement: Additional file 1 — Clinical characteristics of the subjects in the ISOR study. [file 12863_2014_128_MOESM1_ESM.doc]

**Additional file 1.** Clinical characteristics of the subjects in the ISOR study.

| **Parameters** | **ISOR** |  | **Normal weight** | **Overweight** | **Obese** |
| --- | --- | --- | --- | --- | --- |
|  | **study** |  | **Subjects** | **Subjects** | **Subjects** |
|  |  |  |  |  |  |
| N | 787 |  | 340 | 280 | 167 |
| Age (years) | 44.2 ± 10.2 |  | 42.6 ± 10.1 | 44.2 ± 10.2 | 46.3 ± 9.6 |
|  |  |  |  |  |  |
| BMI (kg/m²) | 26.1 ± 5.1 |  | 21.5 ± 2.2 | 27.3 ± 1.4 | 33.5 ± 3.4 |
| Waist circumference (cm) | 87.7 ± 12.4 |  | 77.8 ± 8.1 | 92.0 ± 7.5 | 100.8 ± 9.5 |
| Hip circumference (cm) | 101.9 ± 9.4 |  | 94.3 ± 6.7 | 104.1 ± 5.1 | 113.9 ± 7.8 |
| Waist-to-hip ratio | 0.86 ±0.09 |  | 0.83 ± 0.09 | 0.89 ± 0.08 | 0.89 ± 0.08 |
|  |  |  |  |  |  |

Values are expressed as means ± standard deviation.
