# Supplementary material for: Combined effect of established BMI loci on obesity-related traits in an Algerian population sample
Source: BMC Genet. 2014 Nov 25;15:128. doi: 10.1186/s12863-014-0128-1 (PMC4247883; doi:10.1186/s12863-014-0128-1)
Supplement: Additional file 2 — Individual associations between the 29 genotyped SNPs and anthropometric parameters in the ISOR study. [file 12863_2014_128_MOESM2_ESM.doc]

**Additional file 2**. Individual associations between the 29 genotyped SNPs and anthropometric parameters in the ISOR study.

|  |  |  |  |  |  |  |  |  |  |  |  |  |  |  |  |  |  |
| --- | --- | --- | --- | --- | --- | --- | --- | --- | --- | --- | --- | --- | --- | --- | --- | --- | --- |
|  |  |  | BMI, kg/m² | | |  | Waist circumference, cm | | |  | Hip circumference, cm | | |  | Waist-to-hip ratio | | |
|  |  |  |  |  |  |  |  |  |  |  |  |  |  |  |  |  |  |
|  |  |  |  |  |  |  |  |  |  |  |  |  |  |  |  |  |  |
| Nearby gene or locus | SNP |  |  | SE | *p* |  |  | SE | *p* |  |  | SE | *p* |  |  | SE | *p* |
|  |  |  |  |  |  |  |  |  |  |  |  |  |  |  |  |  |  |
| *FTO* | rs9939609 |  | 0.41 | 0.26 | 0.11 |  | 0.38 | 0.63 | 0.55 |  | 0.67 | 0.51 | 0.19 |  | -0.002 | 0.004 | 0.67 |
| *TMEM18* | rs2867125 |  | 0.38 | 0.33 | 0.25 |  | 0.56 | 0.81 | 0.49 |  | 0.98 | 0.65 | 0.13 |  | -0.005 | 0.005 | 0.38 |
| *MC4R* | rs571312 |  | -0.02 | 0.31 | 0.94 |  | 0.08 | 0.75 | 0.92 |  | -0.19 | 0.60 | 0.75 |  | 0.002 | 0.005 | 0.73 |
| *GNPDA2* | rs10938397 |  | 0.24 | 0.26 | 0.35 |  | 0.41 | 0.64 | 0.52 |  | 0.23 | 0.51 | 0.65 |  | 0.002 | 0.004 | 0.55 |
| *BDNF* | rs10767664 |  | 0.07 | 0.31 | 0.83 |  | 0.48 | 0.76 | 0.53 |  | 0.18 | 0.61 | 0.76 |  | 0.004 | 0.005 | 0.38 |
| *NEGR1* | rs2815752 |  | 0.17 | 0.27 | 0.52 |  | 0.42 | 0.66 | 0.52 |  | 0.24 | 0.53 | 0.65 |  | 0.004 | 0.004 | 0.39 |
| *SH2B1* | rs7359397 |  | 0.22 | 0.35 | 0.53 |  | 1.26 | 0.86 | 0.14 |  | 0.60 | 0.69 | 0.38 |  | 0.006 | 0.006 | 0.28 |
| *ETV5* | rs9816226 |  | 0.31 | 0.30 | 0.30 |  | 0.89 | 0.73 | 0.22 |  | 0.81 | 0.58 | 0.17 |  | -0.001 | 0.005 | 0.85 |
| *MTCH2* | rs3817334 |  | -0.18 | 0.24 | 0.46 |  | -0.15 | 0.58 | 0.80 |  | -0.14 | 0.47 | 0.76 |  | 0.001 | 0.004 | 0.89 |
| *KCTD15* | rs29941 |  | 0.03 | 0.27 | 0.90 |  | -0.17 | 0.67 | 0.81 |  | 0.27 | 0.54 | 0.62 |  | -0.003 | 0.004 | 0.52 |
| *SEC16B* | rs543874 |  | 0.56 | 0.34 | 0.10 |  | 0.86 | 0.82 | 0.30 |  | 1.06 | 0.66 | 0.11 |  | -0.001 | 0.005 | 0.82 |
| *TFAP2B* | rs987237 |  | 0.20 | 0.32 | 0.54 |  | 0.37 | 0.79 | 0.64 |  | 0.17 | 0.64 | 0.79 |  | 0.001 | 0.005 | 0.81 |
| *FAIM2* | rs7138803 |  | 0.44 | 0.27 | 0.10 |  | 1.12 | 0.65 | 0.09 |  | 0.42 | 0.52 | 0.42 |  | 0.007 | 0.004 | 0.09 |
| *NRXN3* | rs10150332 |  | -0.27 | 0.30 | 0.37 |  | -1.19 | 0.73 | 0.10 |  | -0.72 | 0.58 | 0.21 |  | -0.004 | 0.005 | 0.39 |
| *RBJ* | rs713586 |  | 0.62 | 0.25 | 0.01 |  | 1.31 | 0.62 | 0.03 |  | 0.99 | 0.49 | 0.04 |  | 0.004 | 0.004 | 0.29 |
| *GPRC5B* | rs12444979 |  | 0.31 | 0.40 | 0.44 |  | 1.29 | 0.98 | 0.19 |  | -0.27 | 0.78 | 0.73 |  | 0.016 | 0.006 | 0.01 |
| *MAP2K5* | rs2241423 |  | -0.49 | 0.28 | 0.07 |  | -1.61 | 0.68 | 0.02 |  | -1.29 | 0.54 | 0.02 |  | -0.005 | 0.004 | 0.29 |
| *QPCTL* | rs2287019 |  | -0.14 | 0.31 | 0.65 |  | -0.35 | 0.75 | 0.64 |  | 0.00 | 0.60 | 0.99 |  | -0.005 | 0.005 | 0.27 |
| *TNNI3K* | rs1514175 |  | -0.13 | 0.24 | 0.58 |  | -0.03 | 0.59 | 0.95 |  | -0.33 | 0.47 | 0.49 |  | 0.003 | 0.004 | 0.44 |
| *SLC39A8* | rs13107325 |  | -1.21 | 0.85 | 0.15 |  | -4.58 | 2.09 | 0.03 |  | -2.04 | 1.68 | 0.22 |  | -0.031 | 0.014 | 0.02 |
| *FLJ35779* | rs2112347 |  | 0.06 | 0.26 | 0.82 |  | -0.19 | 0.65 | 0.76 |  | 0.53 | 0.52 | 0.31 |  | -0.006 | 0.004 | 0.16 |
| *LRRN6C* | rs10968576 |  | 0.42 | 0.36 | 0.24 |  | 0.47 | 0.88 | 0.60 |  | 0.61 | 0.71 | 0.39 |  | 0.000 | 0.006 | 0.95 |
| *TMEM160* | rs3810291 |  | 0.23 | 0.24 | 0.35 |  | 0.75 | 0.60 | 0.21 |  | 0.28 | 0.48 | 0.56 |  | 0.005 | 0.004 | 0.19 |
| *PRKD1* | rs11847697 |  | 0.17 | 0.36 | 0.64 |  | 0.16 | 0.88 | 0.86 |  | -0.26 | 0.70 | 0.71 |  | 0.006 | 0.006 | 0.29 |
| *LRP1B* | rs2890652 |  | 0.17 | 0.33 | 0.62 |  | 0.67 | 0.82 | 0.42 |  | 0.12 | 0.66 | 0.85 |  | 0.005 | 0.005 | 0.38 |
| *PTBP2* | rs1555543 |  | 0.30 | 0.25 | 0.23 |  | 1.20 | 0.62 | 0.06 |  | 0.51 | 0.50 | 0.31 |  | 0.009 | 0.004 | 0.04 |
| *MTIF3* | rs4771122 |  | 0.25 | 0.32 | 0.42 |  | 0.28 | 0.77 | 0.71 |  | 0.90 | 0.62 | 0.15 |  | -0.004 | 0.005 | 0.37 |
| *RPL27A* | rs4929949 |  | 0.67 | 0.24 | 0.006 |  | 1.27 | 0.59 | 0.03 |  | 1.03 | 0.48 | 0.03 |  | 0.003 | 0.004 | 0.37 |
| *NUDT3* | rs206936 |  | 0.17 | 0.27 | 0.53 |  | 0.64 | 0.65 | 0.33 |  | 0.19 | 0.52 | 0.71 |  | 0.007 | 0.004 | 0.11 |
|  |  |  |  |  |  |  |  |  |  |  |  |  |  |  |  |  |  |

The β coefficients represent the effect sizes. SE: standard error.

*p* values were adjusted for age, gender, physical activity and smoking status.
